# Covariance and Correlation Analysis of Resting State Functional Magnetic Resonance Imaging Data Acquired in a Clinical Trial of Mindfulness-Based Stress Reduction and Exercise in Older Individuals

**DOI:** 10.3389/fnins.2022.825547

**Published:** 2022-03-18

**Authors:** Abraham Z. Snyder, Tomoyuki Nishino, Joshua S. Shimony, Eric J. Lenze, Julie Loebach Wetherell, Michelle Voegtle, J. Philip Miller, Michael D. Yingling, Daniel Marcus, Jenny Gurney, Jerrel Rutlin, Drew Scott, Lisa Eyler, Deanna Barch

**Affiliations:** ^1^Mallinckrodt Institute of Radiology, Washington University School of Medicine, St. Louis, MO, United States; ^2^Department of Neurology, Washington University School of Medicine, St. Louis, MO, United States; ^3^Department of Psychiatry, Washington University School of Medicine, St. Louis, MO, United States; ^4^VA San Diego Healthcare System, San Diego, CA, United States; ^5^Department of Psychiatry, University of California, San Diego, San Diego, CA, United States; ^6^Division of Biostatistics, Washington University School of Medicine, St. Louis, MO, United States; ^7^Master of Social Welfare Program, University of California, Berkeley, Berkeley, CA, United States; ^8^Department of Psychological and Brain Sciences, Washington University, St. Louis, MO, United States

**Keywords:** functional connectivity, covariance, correlation, exercise, mindfulness, resting state—fMRI

## Abstract

We describe and apply novel methodology for whole-brain analysis of resting state fMRI functional connectivity data, combining conventional multi-channel Pearson correlation with covariance analysis. Unlike correlation, covariance analysis preserves signal amplitude information, which feature of fMRI time series may carry physiological significance. Additionally, we demonstrate that dimensionality reduction of the fMRI data offers several computational advantages including projection onto a space of manageable dimension, enabling linear operations on functional connectivity measures and exclusion of variance unrelated to resting state network structure. We show that group-averaged, dimensionality reduced, covariance and correlation matrices are related, to reasonable approximation, by a single scalar factor. We apply this methodology to the analysis of a large, resting state fMRI data set acquired in a prospective, controlled study of mindfulness training and exercise in older, sedentary participants at risk for developing cognitive decline. Results show marginally significant effects of both mindfulness training and exercise in both covariance and correlation measures of functional connectivity.

## Introduction

Evaluation of resting state fMRI functional connectivity (FC) currently is dominated by two methods: Seed-based correlation (SBC) (Shehzad et al., 2009; Zuo et al., 2010) and spatial independent component analysis (sICA) (Beckmann et al., 2005). SBC conventionally is evaluated by Pearson correlation of two time series extracted either from seed regions of interest (ROIs) or voxels, essentially as first described by Biswal et al. (1997). Pearson correlation is a dimensionless, normalized measure that is invariant with respect to signal amplitude. sICA is intrinsically insensitive to signal amplitude. Accordingly, commonly used analysis procedures normalize input time series to unit variance as a preliminary step (e.g., Beckmann et al., 2005; Allen et al., 2011). Concurrently, substantial evidence has accumulated during the past decade indicating that the amplitude of spontaneous blood oxygen level dependent (BOLD) fluctuations is a meaningful indicator of the brain’s functional integrity in psychiatric conditions (Gong et al., 2020), age-related cognitive decline (Vieira et al., 2020), and neurodegenerative diseases (Pan et al., 2017). Moreover, it has been reported that, in healthy individuals, the temporal standard deviation of the BOLD signal (SD_*BOLD*_) indexes cognitive capacity in young as well as older individuals (Garrett et al., 2013; Grady and Garrett, 2014; Pan et al., 2017). This feature of the BOLD signal is ignored in conventional SBC and sICA.

Another issue addressed in this work concerns the dimensionality of the BOLD signal, which is known to be limited (Cordes and Nandy, 2006; Gotts et al., 2020). Functional connectivity (FC) is a second order statistic (Liegeois et al., 2017) that exists in a space of enormous dimension. Thus, given *n* ROIs, there are *n* ⋅ (*n* − 1)/2 unique ROI pairs and a corresponding number of potential FC measures. Thus, for example, if *n* = 300, as in the present work, *n* ⋅ (*n* − 1)/2 = 44,850. One strategy for dealing with this high dimensionality is to restrict the FC analysis to seed ROIs representing one or a very small number of *a priori* selected functional systems. This approach is suitable for testing *a priori* hypotheses concerning particular functional systems or loci within the brain. However, this option does not apply when the objective is to conduct a wholly data-driven, whole-brain FC study. Rational approaches to dealing with the discrepancy between the measured vs. true dimensionality of FC data have not been widely adopted.

Here, we present an approach to the challenge of obtaining whole-brain FC measures that incorporates dimensionality reduction while simultaneously accounting for the amplitude of spontaneous BOLD signal fluctuations. To this end we analyze resting state fMRI data acquired during the course of a large scale, prospective study of mindfulness meditation and exercise in older (age 65–84 years), sedentary participants at risk for developing cognitive decline. This study was conducted by the MEDEX (Mindfulness, EDucation, and EXercise) Research Group, a consortium comprising Washington University in Saint Louis (WUSM) and the University of California in San Diego (UCSD) and is the first study of its kind. It is registered in ClinicalTrials.gov (NCT02665481). A full description of the MEDEX study design is given in Wetherell et al. (2020). The rationale underlying the MEDEX study is that pharmacological treatments that halt or reverse aging-associated cognitive decline are not available. However, substantial evidence indicates that physical exercise ameliorates the manifold negative consequences of aging (Gallaway et al., 2017; Dauwan et al., 2021). Other studies have suggested that behavioral interventions, especially, mindfulness-based stress reduction (MBSR) (Kabat-Zinn et al., 1992) may improve cognitive function and reduce stress or depression in older individuals (Hazlett-Stevens et al., 2019). The question, then, is whether the effects of mindfulness training and exercise are detectable by analysis of resting state BOLD signals.

The methodology described herein was developed, in part, to address this question. We demonstrate novel methodology that addresses the problem of dimensionality in FC analysis while accounting for the amplitude of spontaneous BOLD signal fluctuations. We report resting state functional magnetic resonance imaging (rs-fMRI) outcomes derived from the MEDEX study. Non-neuroimaging outcomes of the MEDEX study will be reported elsewhere.

## Materials and Methods

### Participants and Study Design

Participants were recruited at two-sites, Washington University in Saint Louis (WUSM) and University of California San Diego (UCSD). Inclusion criteria included age 65–84 years, sedentary lifestyle, self-reported cognitive complaints but non-demented cognitive status, and no contraindication to magnetic resonance imaging (MRI), e.g., metal implants. Participants with biomarker evidence of preclinical Alzheimer’s disease were not excluded. All participants gave written informed consent and received no remuneration. The IRB committees at WUSM and UCSD provided oversight over all aspects of the study.

Participants were randomly assigned to one of 4 interventions for an 18-month period, according to a 2 × 2 factorial design: (i) MBSR-only: Weekly instructor-led MBSR group-based classes for 10 weeks and then monthly booster sessions; (ii) Exercise-only: Twice-weekly instructor-led exercise group classes, including aerobic, strength, and functional training, for 6 months and then weekly booster session; (iii) MBSR + exercise: Both MBSR sessions and exercise sessions; (iv) Health education: instructor-led sessions with health education content which included neither MBSR nor exercise and which matched the MBSR condition in session frequency and time. Participants were also instructed to practice at home over the entire 18-month duration of the study. The goal of home practice was up to 45 min daily mindfulness practice in the MBSR condition and 150 min/week exercise in that condition. MRI scanning was performed at baseline (before any intervention), at 6 months, and at 18 months.

### Magnetic Resonance Imaging Acquisition

Two Siemens (Erlangen Germany) scanners equipped with 20-channel head coils were used at WUSM. At UCSD, MRI was acquired using a GE MR750 3T MRI scanner (GE, Milwaukee, WI) equipped with an 8 Channel head coil (Table 1). Structural imaging included T1-weighted (WUSM MP-RAGE; TR = 2,400 ms, TE = 3.16 ms, TI = 1,000 ms; 1 × 1 × 1 mm voxels) (UCSD MPRAGE; TE = 3.036, TI = 1000 ms; and 1 × 1 × 1 mm voxels) and T2-weighted (WUSM SPACE; TR = 3,200 ms, TE = 458 ms; 1 × 1 × 1 mm voxels) (UCSD CUBE; TR = 2,500, TE = 73.37 ms; 1 × 1 × 1 mm voxels) anatomical images. Resting state fMRI (rs-fMRI) was acquired with a multi-echo sequence (WUSM TR = 2,960 ms, TE = 15, 31.3, 47.6, 63.9 ms; 4 × 4 × 4 mm voxels) (UCSD TR = 2,740 ms, TE = 14.8, 28.4, 42, 55.6; 4 × 4 × 4 mm voxels) including 140 frames (volumes) per run. Up to 4 rs-fMRI runs were obtained in each session. During rs-fMRI acquisition, participants were shown a silent video of neutral content (relaxing nature scenes) and were instructed to keep their head still, stay awake, and not meditate. To simplify statistical comparisons, the present analysis includes only participants who completed all 4 resting state fMRI runs (23.3 min total WUSM, 25.6 min total UCSD) in all three scanning sessions. In accordance with the longitudinal experimental design, each participant was scanned with the same scanner during all three visits. All MRI scans were conducted at least 48 h after the participant’s most recent exercise session (in class or at home) to avoid acute exercise effects in scan findings. Demographic information broken down by scanner is listed in Table 2. Cognitive performance and adherence data for the 4 intervention groups are listed in Table 1.

**TABLE 1:** Characteristics of participants (*N* = 315) contributing to the results (Table 4) broken down by treatment group.

	MBSR	Exercise	MBSR + +Exercise	Health Ed
*N*	105	95	91	84
Cognitive score	93.2	93.3	92.7	92.5
Adherence (%)	81.6	77.4	81.8, 72.9	76.7

*Reported values are treatment group means. Cognitive Score is the normed (mean = 100, SD = 15), Fluid Cognition Composite test score from the NIH Toolbox Cognition Battery (Heaton et al., 2014), measured at baseline. Adherence is% classes attended over the study duration. MBSR and Health Education classes were once weekly for 10 weeks, then once monthly (total = 100 classes over 18 months). Exercise classes were twice weekly for 6 months, then once weekly (total = 25 over 18 months).*

**TABLE 2:** MEDEX participants broken down by scanner.

Institution	Scanner	Ntot	Nret	Age ± SD	%Female	%Frames retained
San Diego	GE Discovery MR 750	195	187	71.4 ± 4.7	80%	82.1%
WUSM Bay3	Siemens Prisma Fit	166	150	71.3 ± 4.9	76%	84.4%
WUSM CCIR	Siemens Tim Trio	39	38	69.6 ± 3.5	76%	83.8%
total		400	375	71.2 ± 4.7	78%	83.2%

*Listed age refers to the baseline session. Ntot is participants scanned in all 3 sessions. Nret is participants contributing to the Results, retained after exclusion owing to excessive head motion in any session. %frames retained refers to data contributing to the Results after motion “scrubbing” (see below).*

### Resting State Functional Magnetic Resonance Imaging Processing

Pre-processing largely following methods described by Raut et al. (2019). Initial preprocessing was computed on data summed over all echoes and included rigid body correction for head motion within and across fMRI runs, correction of bias field inhomogeneities using the FAST module in FSL (Zhang et al., 2001), and slice timing correction. Atlas transformation was computed by 12-parameter affine registration of the structural T1w images to composition of affine transforms linking the fMRI data (head motion corrected functional frame average) to the atlas-representative target image (711-2B version of Talairach space). A scanner-specific target was generated for each of the three scanners (Buckner et al., 2004) to eliminate systematic atlas transform differences arising from variable T1w contrast. Transforms linking the functional data to the atlas representative target via the structural images were composed (fMRI→T2w→T1w→ atlas) and then applied in one-step to resample the functional data (4 echoes per frame) in atlas space (3 mm^3^ voxels).

The atlas-transformed, multi-echo data were modeled according to standard theory (Poser et al., 2006) in which reconstructed image intensity depends mono-exponentially on TE. Thus,


(1) $$S\left(TE\right)=S_{0}e\text{xp}\left(-R_{2}^{*}TE\right),$$

where *S* is intensity and *S*_0_ is intensity extrapolated to *TE* = 0. *S*_0_ and $R_{2}^{*}$ are free parameters determined on the basis of multiple echoes (4 in this case). *S*_0_ and $R_{2}^{*}$ were estimated according to Eq. 1 separately for every voxel and frame using log-linear fitting. Empirical evidence (Power et al., 2018) shows that fluctuations in the value of *S*_0_ primarily reflect spin history artifacts generated by head motion (Friston et al., 1996), whereas $R_{2}^{*}$ reflects BOLD contrast (Ogawa et al., 1992) as well as changes in arterial pCO_2_ (Birn et al., 2006). Accordingly, frame-to-frame variability in *S*_0_ was eliminated by replacing time-dependent values with the (voxel-wise) fMRI run temporal average. The multi-echo modeling procedure then evaluated Eq. 1 at the TE corresponding to the second echo (31.3 ms for WUSM, 28.4 ms for UCSD) and output a volumetric time series that we here designate “Sfit.”

**FIGURE 1:**
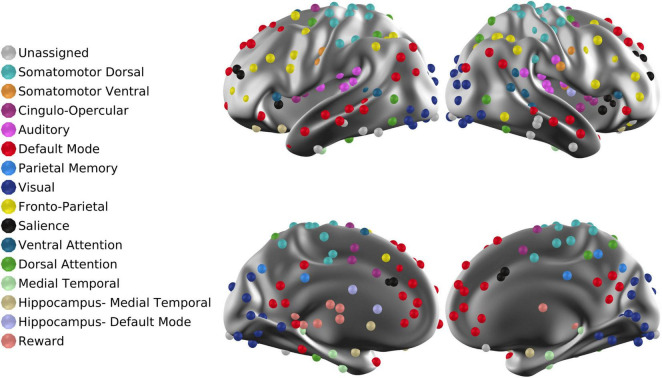
ROIs projected onto the cortical surface. The total number of ROIs is 300. Each ROI is associated with one of 16 RSNs. “Unassigned” refers to regions of the brain in which fMRI signals are unreliable owing to susceptibility dropouts.

The Sfit volumetric time series acquired over 4 runs in each session were virtually concatenated. Next, to enable interpretation of fMRI signal fluctuation on an absolute scale, the functional data were intensity normalized (one scalar multiplier) to obtain a whole-brain mode value of 1,000. Thus, following mode-1,000 intensity normalization, a voxel-wise temporal standard deviation of 10 corresponds to 1% rms signal modulation. Denoising began by marking frames for subsequent exclusion from the FC computations by reference to the DVARS timeseries, i.e., root-mean-square inter-frame intensity changes (Smyser et al., 2010; Power et al., 2014). The frame censoring criterion was adjusted on a per-session basis to accommodate baseline shifts in the DVARS measure (White et al., 2020). Frame censoring statistics are included in Table 2. The concatenated data then were spatially filtered (6 mm FWHM in each cardinal direction) and temporally filtered (demeaned, detrended, low-pass cut-off at 0.1 Hz). Additional denoising was accomplished by regression of timeseries using a strategy similar to CompCor (Behzadi et al., 2007). Image-derived nuisance regressors were extracted from FreeSurfer 6.0.0-segmented regions (Fischl, 2012) following co-registration with the functional data in atlas space. Nuisance regressors included (i) six rigid body parameter time series derived from within-run head motion correction; (ii) image-derived regressors extracted from multiple sub-regions within three anatomical compartments: white matter, ventricles, and the extracranial cerebrospinal fluid (CSF); (iii) the global signal averaged over the whole brain (Fox et al., 2009; Power et al., 2017; Ciric et al., 2018). Image-derived nuisance regressors were dimensionality reduced by PCA as previously described (Raut et al., 2019). The final number of nuisance regressors used to denoise the Sfit data varied according to the quality of the data (mean = 38, SD = 13, max = 85, min = 9). To account for the effects of the video stimuli shown during fMRI acquisition, the mean session-specific video fMRI response averaged over all participants was subtracted from each participant’s timeseries. The preprocessed and denoised timeseries was extracted from 300 functionally defined brain regions of interest (ROIs) (Seitzman et al., 2020; Figure 1), and pairwise region of Interest (ROI) correlation values were computed, omitting frames previously marked for censoring.

### Dimensionality Reduction and Derivation of Fixed Basis

The dimensionality of whole brain resting state fMRI data (number independent signals distinguishable from noise) is limited (Cordes and Nandy, 2006; Gotts et al., 2020). Accordingly, it is possible that dimensionality reduction may enhance sensitivity to experimental interventions by removing variance unrelated to resting state network (RSN) structure. Here, dimensionality reduction was effected by proper orthogonal decomposition (POD), an analytic technique closely related to principal component analysis (PCA) (Liang et al., 2002). We refer to this method as POD to emphasize that the objective is to derive a basis of reduced dimensionality on which to represent a high dimensional process. We retained the top 20 components, which is comparable to the number of non-noise components identified in prior work (Allen et al., 2011). Let *X_i_* represent the fMRI data where *i* indexes a particular session of a particular participant. *X*_*i*_ is *m* × *L_i_*, where *m* is the number of ROIs (300 in the present work) and *L*_*i*_ is number of rs-fMRI samples (total length of resting state data excluding censored frames) in session *i*. The covariance matrix of *X*_*i*_ is


(2) $$C_{i}=\left(1/L_{i}\right)X_{i}X_{i}^{T}.$$

The mean covariance matrix in the studied cohort is $\bar{C}=\left(1/\text{N}\right)\sum_{i}C_{i}$, where *N* is the total number of sessions. POD of $\bar{C}$ yields


(3) $$\bar{C}=W\text{Λ}W^{T},$$

where the eigenvectors of *C* constitute the columns of *W* and Λ is a diagonal matrix of eigenvalues. The dimensionality reduced mean covariance matrix, *Ĉ*, is obtained by truncating Λ, retaining the left upper 20 × 20 submatrix, $\hat{\text{Λ}}$. Thus,


(4) $$\hat{C}=\hat{W}\hat{\text{Λ}}{\hat{W}}^{T}.$$

Thus, *Ŵ* constitutes a fixed basis of reduced dimensionality that provides a means of representing the covariance structure of all participants in a canonical format.

Left multiplying *X*_*i*_ by *Ŵ^T^* yields ${\hat{Y}}_{i}={\hat{W}}^{T}X_{i}$, the projection of session *i*’s fMRI data onto the fixed basis. The covariance matrix of this projection is


(5) $$\left(1/L_{i}\right){\hat{Y}}_{i}{\hat{Y}}_{i}^{T}=\left(1/L_{i}\right){\hat{W}}^{T}X_{i}X_{i}^{T}\hat{W}={\hat{W}}^{T}C_{i}\hat{W},$$

where *C*_*i*_ is the full covariance matrix of session *i*. POD of this matrix yields $C_{i}=W_{i}{\text{Λ}}_{i}W_{i}^{T}$. The first 20 eigenvalues of *C*_*i*_ are the diagonals of ${\hat{W}}_{i}^{T}C_{i}{\hat{W}}_{i}={\text{Λ}}_{i}^{\prime }$. Ideally, if all *X*_*i*_ shared the identical eigenstructure, i.e., if ${\hat{W}}_{i}=\hat{W}$ for all *i*, then we could write $\left(1/L_{i}\right){\hat{Y}}_{i}{\hat{Y}}_{i}^{T}={\hat{W}}^{T}{\hat{W}}_{i}{\hat{\text{Λ}}}_{i}^{\prime }{\hat{W}}_{i}^{T}\hat{W}={\hat{\text{Λ}}}_{i}^{\prime }={\hat{\text{Λ}}}_{i}$. In practice, this identity is only approximate $\left({\hat{W}}_{i}\approx \hat{W}\right)$. Nevertheless, we *define* the diagonal entries of $\left(1/L_{i}\right){\hat{Y}}_{i}{\hat{Y}}_{i}^{T}\equiv {\hat{\text{Λ}}}_{i}$ as the estimated magnitude of 20 covariance components in session *i* and *define* ${\hat{C}}_{i}\equiv \hat{W}{\hat{\text{Λ}}}_{i}{\hat{W}}^{T}$ as the projection of session *i*’s covariance structure onto the fixed basis. The extent to which the $diag\left(1/L_{i}\right){\hat{Y}}_{i}{\hat{Y}}_{i}^{T}$ is equivalent to the first 20 eigenvalues of *C*_*i*_ is the extent to which ${\hat{W}}^{T}{\hat{W}}_{i}$ is equal to the 20 × 20 identity matrix, *I*. Similarity of subgroup eigenstructure is reported in Supplementary Figure 1.

Importantly, ${\hat{\text{Λ}}}_{i}$ and ${\hat{C}}_{i}=\hat{W}{\hat{\text{Λ}}}_{i}{\hat{W}}^{T}$ are informationally equivalent (because *Ŵ* is fixed). Hence, $\left\{{\hat{\text{Λ}}}_{i}\right\}$ can be subjected to algebraic operations, e.g., averaging over participant subgroups and inputting into linear regressions. The results of these operations are linear combinations of $\left\{{\hat{\text{Λ}}}_{i}\right\}$ which can be inserted into the form of Eq. 4 and displayed as covariance matrices. For notational simplicity, define ${\text{Ψ}}_{i}={\left(diag\left({\hat{\text{Λ}}}_{i}\right)\right)}^{T}.$ Thus, ${\text{Ψ}}_{i}$ represents the 20 covariance components corresponding to a particular visit of a particular participant reshaped as a 1 × 20 row vector. Let ${\text{Ψ}}_{*}$ denote some linear combination of $\left\{{\text{Ψ}}_{i}\right\}$; then there exists a one-to-one correspondence between ${\text{Ψ}}_{*}$ and ${\hat{C}}_{*}=\hat{W}{\hat{\text{Λ}}}_{*}{\hat{W}}^{T}$. The asterisk in the preceding expression denotes any particular subgroup (e.g., participants in the exercise group imaged at 6 months). We present results using both ${\hat{C}}_{*}$ and ${\text{Ψ}}_{*}$ representations. To equalize total BOLD power over scanners, the individual covariance matrices were scaled by a site-specific factor ensuring that the traces of site-specific mean covariance matrices were equal (see Supplementary Figure 2). Further, site-specific contributions to ${\text{Ψ}}_{i}$ were removed by linear regression.

Dimensionality reduction and basis definition for correlation (as opposed to covariance) FC are essentially similar. Thus, let $\bar{r}$ be the mean correlation matrix averaged over all participants and sessions. Then


(6) $$\hat{r}=\hat{w}\hat{\lambda }{\hat{w}}^{T}$$

is the dimensionality reduced mean correlation matrix and *ŵ* contains eigenvectors defining the correlation FC basis. Equation 6 is analogous to Eq. 4. The remainder of the above-discussed considerations, in particular, projection of individual FC components onto a fixed basis (Eq. 5), apply equally well to the case of correlation FC. Table 3 lists all variables used in the statistical evaluation and presentation of results.

**TABLE 3:** Symbols referring to measured quantities in statistical testing and presentation of results.

	Covariance	Correlation	Dimension
Full dimensionality FC matrix	*C*	*r*	300 × 300
Diagonal eigenvector matrix of *C*	Λ	λ	300 × 300
Basis derived by POD of control group FC matrices	$\hat{W}$	$\hat{w}$	300 × 20
Dimensionality reduced FC matrix	$\hat{C}$	$\hat{r}$	300 × 300
Component magnitudes derived by projection on basis	Ψ	P	1 × 20
$\hat{C}$: $\hat{r}$global scalar proportionality	υ		Scalar
$\hat{C}\cdot \hat{r}$ Pearson correlation over block averages	η		Scalar

### Statistical Testing

Following dimensionality reduction, the covariance and correlation measures obtained in a particular session are represented as ${\text{Ψ}}_{i}$ and P_*i*_, respectively. To define additional nomenclature by example, let ${\text{Δ}}^{visit2-1}$ represent a longitudinal change operator. Thus, ${\text{Δ}}^{visit2-1}{\text{Ψ}}_{MBSR}={\text{Ψ}}_{MBSR}^{visit2}-{\text{Ψ}}_{MBSR}^{visit1}$ represents the mean longitudinal change in covariance in participants with mindfulness training. Similarly let ${\text{Δ}}_{MBSR-noMBSR}$ represent a treatment contrast operator. Quantities of experimental interest include


(7) $${\text{Δ}}_{MBSR-noMBSR}{\text{Δ}}^{visit2-1}\text{Ψ},$$

i.e., the effect of mindfulness training on longitudinal change in covariance FC over the first 6 months, and similar expressions formulated in terms of P (correlation component magnitudes). We took the *L*_1_ norm (sum of absolute values) of these 1 × 20 quantities as the measure of interest. Statistical significance of quantities of the form represented by Eq. 7 was assessed by permutation resampling over 10,000 repetitions. Thus, the link between participant and treatment was randomly shuffled, maintaining constant treatment group sizes, and the distribution of *L*_1_ norm values compiled over repetitions. The likelihood of observing the true experimental outcome by chance then corresponds to a particular percentile of the surrogate distribution.

### Covariance:Correlation Matrix Global Proportionality

**FIGURE 2:**
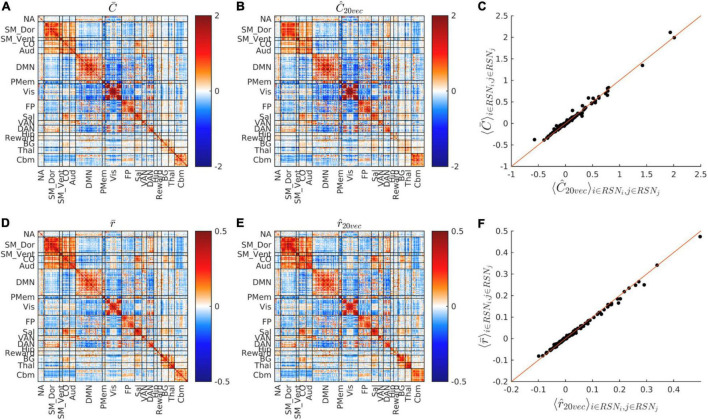
FC matrix dimensionality reduction. **(A,B)** Show $\bar{C}$ and *Ĉ*, i.e., the cohort-average covariance FC matrix before and after dimensionality reduction. **(C)** Shows the scatter plot of $\bar{C}$ vs. *Ĉ* matrix block means, i.e., ${\bar{C}}_{k}$ vs. *Ĉ_k_* (see section “Materials and Methods”). **(D,E)** Show the cohort-average, conventional Pearson correlation FC matrix before and after dimensionality reduction, i.e., $\bar{r}$ and $\hat{r}$. **(F)** Shows the ${\bar{r}}_{k}$ vs. ${\hat{r}}_{k}$ scatter plot. Dimensionality reduction preserves almost all RSN structure of both covariance and correlation FC matrices. Note different scales in **(D–F)** vs. **(A–C)**.

As will shortly be shown, *Ĉ* and $\hat{r}$ evaluated over the full dataset exhibit strikingly similar “matrix topographies” (see section “Results”), i.e., differ, to a good approximation, by only a scalar factor. This relation can be symbolically represented as $\hat{C}\approx \upsilon \cdot \hat{r}$. where υ is a scalar factor. We fit this model by minimizing error over matrix block averages. Thus, we minimize $\varepsilon^{2}=\sum_{k}{\left[\langle \hat{C}\rangle_{k}-\upsilon \langle \hat{r}\rangle_{k}\right]}^{2}$, where subscript *k* indexes matrix blocks and the bracket notation denotes averaging over entries within block. These blocks (delineated by heavy lines in Figure 2) are square on the diagonal and rectangular off the diagonal. The “blockwise average” approach to evaluating υ follows from the demonstration that dimensionally reduced FC matrices retain almost all RSN structure. The ordinary least squares estimate for υ is $\sum_{k}{\hat{\langle C\rangle }}_{k}\langle \hat{r}\rangle_{k}/\sum_{k}\langle {\hat{r\rangle }}_{k}^{2}$. The Pearson correlation between *Ĉ* and $\hat{r}$ block averages is $\eta =\sum_{k}{\hat{\langle C\rangle }}_{k}\langle {\hat{r\rangle }}_{k}/{\left[\sum_{k}\langle {\hat{C\rangle }}_{k}^{2}\sum_{k}\langle {\hat{r\rangle }}_{k}^{2}\right]}^{\frac{1}{2}}$ (here, η denotes Pearson correlation to avoid overloading the symbol *r*, which refers to fMRI signal correlations). As is true of Pearson correlation generally, the fraction of total variance accounted for by the global covariance:correlation proportionality model is η^2^. Theoretically, the value of υ depends sequence details as some sequences could weight white matter, gray matter and CSF differently. However, the impact of such dependencies likely are minor as the present methodology includes variance equalization across scanners (see Supplementary Figure 2).

## Results

### Dimensionality Reduction

Figure 2 shows the whole-cohort, mean covariance and correlation matrices before ($\bar{C},\bar{r}$) and after ($\hat{C},\hat{r}$) projection onto their respective fixed bases. The block structure of the matrices, shown in Figure 2 replicates established findings reported in multiple rs-fMRI studies (Laumann et al., 2015; Gotts et al., 2020; Seitzman et al., 2020). It is visually evident that the block structure of these matrices is nearly unaffected by dimensionality reduction. The squared Pearson correlation between ${\bar{C}}_{k}$ and *Ĉ_k_* is 0.985. The squared Pearson correlation between ${\bar{r}}_{k}$ and ${\hat{r}}_{k}$ is 0.996. Thus, dimensionality reduction preserves the block structure of both covariance and correlation FC matrices. At the same time, projection accounts for only 38.7% of total variance in $\bar{C}$ and 36.5% of total variance in $\bar{r}$. Thus, variance outside the fixed bases resides almost entirely within subcomponents of RSNs.

### Anatomical Topography of Covariance and Correlation Basis Vectors

**FIGURE 3:**
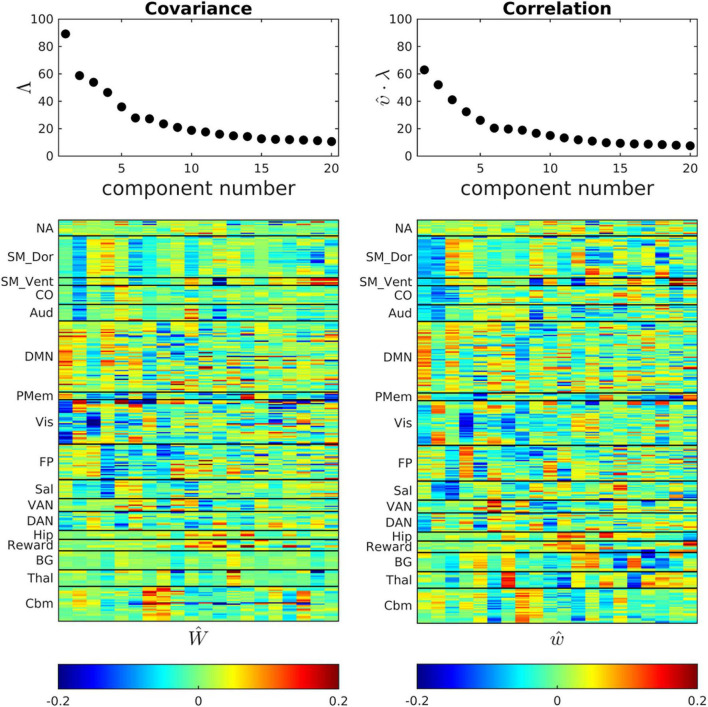
*Ĉ* and $\hat{r}$ bases (eigenvectors) obtained by POD of the cohort-mean covariance and correlation FC matrices. Scree plots are shown above corresponding eigenvectors. The correlation eigenvectors have been multiplied by the scalar constant (υ = 3.67) that minimizes error in the global, block-wise proportionality model, ${\hat{C}}_{k}=\upsilon \cdot {\hat{r}}_{k}$. The first few eigenvectors exhibit clustered, large magnitude loadings within resting state networks, e.g., the DMN. Network structure becomes fragmented at higher eigenvector indices in both the covariance and correlation representations of FC.

Figure 3 shows scree plots and eigenvectors (ROI weights) corresponding to *Ĉ* and $\hat{r}$. The ROI weights in the basis vectors reflect the organization of major functional systems. Thus, the first component of both *Ŵ* and *ŵ* is dominated by the DMN and the second component is dominated by somatomotor dorsal, somatomotor ventral, and cingulo-opercular networks (SM_Dors, SM_Vent, Co.). Higher components exhibit progressively less RSN structure. This loss of RSN structure, coupled with asymptotically small eigenvalues as component indices approach 20, implies that the dimensionality reduction largely preserves meaningful variance in the original data. This point is addressed also in Supplementary Figure 1, which shows that the fixed bases reasonably well represent the correlation structure of participant subgroups.

### Global Covariance: Correlation Proportionality

**FIGURE 4:**
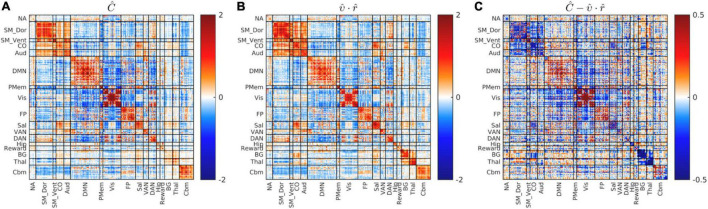
Global blockwise scalar proportionality between the cohort-mean covariance and correlation matrices. **(A)** shows *Ĉ*. **(B)** Shows $\upsilon \cdot \hat{r}$. **(C)** Shows the difference, $\hat{C}-\upsilon \cdot \hat{r}$, on a scale of increased gain. Focal deviations from global blockwise scalar proportionality. These deviations account for only 13% of total variance. Scale change in **(C)**.

Figure 4 shows the dimensionality reduced covariance and correlation matrices corresponding to the whole study cohort. It is evident that these matrices exhibit strikingly similar “matrix topographies.” Moreover, the covariance-FC and correlation-FC basis vectors are similar (see Supplementary Figure 3). Blockwise fitting the model, $\hat{\text{C}}=\upsilon \cdot \hat{r}+\epsilon$, yielded a global covariance:correlation ratio (υ) of 3.67. The proportion of model-consistent variance (η^2^) is 0.866. Figure 4C shows the difference, $\hat{C}-\upsilon \cdot \hat{r}$, i.e., focal deviations from global covariance:correlation proportionality. Such deviations include somewhat greater covariance:correlation ratios in parts of Visual network and the DMN and somewhat lesser ratios in somatomotor and cingulo-opercular cortex. Although these deviations are potentially of physiological interest, they are quantitatively minor (13%). Hence, further consideration of these focal deviations from global proportionality is deferred to future work.

### Effects of MEDEX Interventions on Covariance and Correlation Functional Connectivity

**FIGURE 5:**
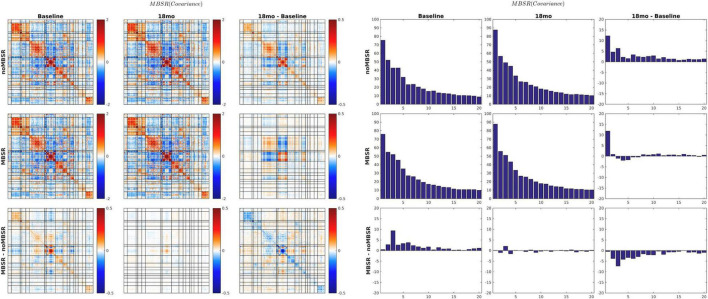
18-months vs. baseline effect of mindfulness training on covariance FC. Covariance matrices and corresponding covariance component bar plots are shown on the left and right of the figure. The information content of the matrix and bar plot displays is identical. At 18 months vs. baseline, all participants, with or without MBSR training, showed increased activity in visual cortex and the DMN with more pronounced VIS-DMN negative covariance (upper matrices in right column). The 18-month change specific to MBSR was less focal and quantitatively modest (lower matrix in right column). In the notation of Eq. 7, the lower right bar plot represents ${\text{Δ}}_{MBSR-noMBSR}{\text{Δ}}^{vist3-1}\text{Ψ}$. Statistical testing of $\left|{\text{Δ}}_{MBSR-noMBSR}{\text{Δ}}^{vist3-1}\text{Ψ}\right|$ is illustrated in Figure 7A (*p* = 0.03).

**FIGURE 6:**
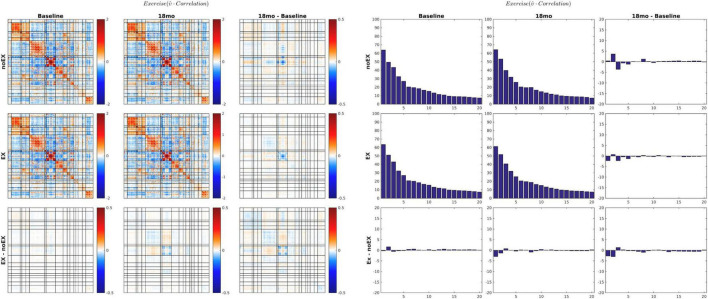
18-months vs. baseline effect of exercise on correlation FC. The format of this figure is identical to Figure 5. As in Figure 6, the information content of the matrix and bar plot displays is identical. Statistical testing of the lower right bar plot is illustrated in Figure 7B.

**FIGURE 7:**
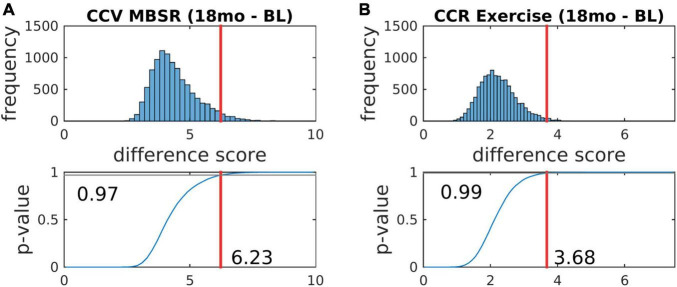
Statistical testing of the effects of mindfulness training and exercise on FC by permutation resampling (10,100 surrogate samples). **(A,B)** Correspond, respectively, to Figures 5, 6. The red lines indicate the likelihood that the actual experimental outcome arose by chance. *p*-values corresponding to other contrasts are reported in Table 4.

The MEDEX study design includes two interventions (MBSR vs. no MBSR) × (Exercise vs. no Exercise) and three visits (Baseline, 6 months, 18 months), which generates 6 potential Intervention × Visit contrasts. We elected to simplify the analysis by focusing on first-order effects of the two types of intervention. Thus, study group comparisons were (1) exercise vs. no exercise (i.e., the exercise-only and MBSR + exercise groups, vs. the MBSR-only and Health Education groups), and (2) MBSR vs. no MBSR (i.e., the MBSR-only and MBSR + exercise groups, vs. the exercise-only and Health Education groups). Display of both covariance and correlation matrix results corresponding to all potential contrasts is not feasible. However, matrix results and component magnitude differences corresponding to contrasts yielding statistically significant results (*p* < 0.05, uncorrected) are shown in Figures 5, 6. The matrix and component magnitude displays are arranged in a 3 × 3 array with contrast over time in columns and contrast over intervention in rows. Figure 5 shows the effect of mindfulness training on covariance FC change at 18 months vs. baseline. Figure 6 shows the effect of exercise on correlation FC change at 18 months vs. baseline. Permutation testing of these effects is illustrated in Figure 7. Summary statistics covering all 6 condition contrasts for both covariance and correlation FC are listed in Table 4.

**TABLE 4:** Significance testing of longitudinal effects of MBSR and exercise on covariance and correlation FC measures.

	6 months–baseline		18 months–baseline		18 months–6 months	
	$\sqrt{\left|\Delta \Psi \right|}$	*p*-value	$\sqrt{\left|\Delta \Psi \right|}$	*p*-value	$\sqrt{\left|\Delta \Psi \right|}$	*p*-value
MBSR	4.93	0.20	6.23	0.03	4.78	0.25
Exercise	4.55	0.30	5.56	0.09	4.18	0.49
	$\sqrt{\left|\Delta \text{P}\right|}$	*p*-value	$\sqrt{\left|\Delta \text{P}\right|}$	*p*-value	$\sqrt{\left|\Delta \text{P}\right|}$	*p*-value
MBSR	2.26	0.35	2.54	0.24	2.46	0.19
Exercise	1.21	0.98	3.68	0.01	3.18	0.03

*|ΔΨ| and |ΔP| denote the L_1_ norm of quantities defined in Eq. 7. Squares roots were evaluated as this modification yielded a more normal (less right-skewed) distribution of permutation resampling surrogate values (see Figure 7). Red and blue highlight indicate p-values significant at p < 0.05 and p < 0.10, respectively, not corrected for multiple comparisons.*

## Discussion

We report FC results obtained with novel methods combining conventional Pearson correlation FC with methodology that preserves BOLD amplitude information. This is the first large scale, prospective study of the effects of mindfulness training and exercise on resting state BOLD fMRI. In the present data, some effects of these interventions are formally significant (*p* < 0.05), omitting correction for multiple comparisons (Table 4). With correction for multiple comparisons (6 tests), these results are properly viewed as marginally significant.

Several prior resting state fMRI studies concerned with the effects of MBSR or similar practices have reported increased conventional (Pearson correlation) FC between dorsolateral prefrontal cortex (dlPFC) and an array of other regions of the brain (Creswell et al., 2016; Taren et al., 2017; Kral et al., 2019). Similarly, exercise or aerobic fitness has been associated with increased (conventionally assessed) FC affecting a variety of widely distributed region pairs (Voss et al., 2010; McGregor et al., 2018). We did not specifically attempt to replicate those findings. However, the present evidence (Figure 6 and Table 4) suggests that the effects of exercise on conventional Pearson correction FC are exceedingly subtle and largely confined to areas of the cerebral cortex concerned with vision. Failure to replicate previously reported effects of exercise on FC measures has been explicitly noted before (Flodin et al., 2017).

Small participant samples undoubtedly account, in part, for replication failure. Sample sizes in all of the above-cited reports, except (Flodin et al., 2017), were a full order of magnitude smaller than the present one (Table 2). However, we suggest that principal challenge in data-directed functional connectivity studies is not fundamentally a matter of sample size. The fundamental problem is the dimensionality of the data space, which encompasses pairs of regions, numbering on the order of 10^4^ in whole-brain studies utilizing dense spatial coverage (see Introduction). In practice, very little prevents an investigator focusing on selected region pairs, after which significant findings may emerge even after seemingly appropriate multiple comparisons correction. Dimensionality reduction offers a means of projecting whole-brain FC measures onto a space of manageable dimension. The investigator may vary the number components retained in the analysis but this does not bias the results provided that the eliminated components exhibit little evidence of structure (see Figure 3). Importantly, the dimensionality of resting state fMRI data is considerably smaller than the space of all densely sampled ROI pairs. This point has been made before (Cordes and Nandy, 2006; Gotts et al., 2020); it is here demonstrated in Figures 2, 3.

The present approach to the representation of FC by projection onto a fixed basis represents a greatly simplified version of previously published methodology (Madsen et al., 2017). In the present data, less than half (38.7%) of all BOLD variance is structured according to RSNs. This means that more than half is unstructured. Much of this unstructured variance arises from electronic noise (Liu, 2016). This variance enters into evaluations of seed-based FC but is not organized at the systems-level, hence, depresses measured correlations. Dimensionality reduction eliminates variance not organized at the systems level, hence, avoids this problem. Additionally, projection of covariance and correlation measures onto a fixed basis provides a straightforward means of regressing out unwanted sources of variance, e.g., scanner dependencies.

Parallel evaluation of covariance FC together with correlation FC is motivated by substantial evidence indicating that the amplitude of spontaneous BOLD fluctuations indexes cognitive capacity (Grady and Garrett, 2014; Pan et al., 2017). For example, it has been shown that BOLD signal variability is a correlate of age-related cognitive decline (Vieira et al., 2020). Moreover, it has been reported that the amplitude of spontaneous BOLD signal fluctuations correlates with performance measures, independently in young as well as older individuals (Garrett et al., 2013). Prior relevant work suggests that the amplitude of low frequency fluctuations (ALFF) is altered in adult long-term meditators (Berkovich-Ohana et al., 2016). More specifically, it has been reported that mindfulness or “mind-body” training *decreases* ALFF in the default mode network (DMN) (Berkovich-Ohana et al., 2016; Yang et al., 2019) or hippocampus (Tao et al., 2019). [The hippocampus is closely linked to the DMN (Vincent et al., 2007)]. Such decreases in ALFF are broadly consistent with the results shown in Figure 5. Thus, decreased ALFF may not necessarily be inconsistent with a positive influence of MBSR on performance measures or mood. At the same time, focal ALFF increases, particularly in networks associated with cognitive control, have been reported as a correlate of mindfulness training (Wei et al., 2017), which is consistent with the work of Grady and Garrett (2014).

The quantity previously reported as SD_*BOLD*_ (Garrett et al., 2013) or ALFF (Wei et al., 2017) is the square root of the quantity appearing on the diagonal of covariance FC matrices. Thus, covariance FC effectively comprises SD_*BOLD*_/ALFF but also broadens the analysis to include cross-RSN interactions which appear in off-diagonal blocks. Other laboratories have reported BOLD fMRI signal covariance matrices (Varoquaux et al., 2010). However, computation of BOLD fMRI covariance FC is hardly at all represented in the extant literature (but see Smyser et al., 2016). The present demonstration of approximately uniform global covariance:correlation proportionality (υ; Figure 4) is novel. This result implies that the amplitude of spontaneous BOLD fluctuations is approximately uniform over the cortical surface. Although the impact of MBSR on covariance FC was modest in the present data, Figure 5 shows covariance component magnitude changed over the 18 months between visits 1 and 3, unrelated to treatment. Specifically, BOLD fluctuations increased primarily in visual cortex and, to a lesser extent, the DMN, with concomitant strengthening of VIS:DMN negatively signed covariance. Much of this change is attributable to an increase in the magnitude of the first covariance component (see Figure 3). Conceivably, this change may reflect different videos played at visits 1 and 3, although the movie-evoked response was removed from the BOLD data before any FC computations. An explanation for this finding is not readily apparent. However, it does suggest that covariance FC (as well SD_*BOLD*_/ALFF) may be subject to variability owing to as yet poorly understood factors.

## Conclusion

The principal objectives of this work are demonstration of the computational advantages of dimensionality reduction in the analysis of resting state fMRI data and introduction of parallel covariance/correlation analysis of functional connectivity. These advantages include (i) projection of FC measures, which, in “raw” form, exist in a space of very large dimension, onto a space of manageable dimension; (ii) enabling exclusion of unwanted sources of variance (e.g., scanner dependencies) using simple linear regression; (iii) exclusion from FC computations of unstructured variance that otherwise would bias FC measures toward zero; (iv) introduction of the global covariance:correlation proportionality constant, υ. Although the value of υ was unaffected by any of the present experimental manipulations, this ratio potentially carries physiological significance and may be revealing in other experimental contexts. Application of the novel methodology to the present data set revealed only marginally significant effects of both mindfulness training and exercise, in contrast to prior reports. In view of the unprecedentedly large participant sample relative to related prior work, this outcome raises questions concerning the replicability of prior findings.

## Data Availability Statement

The raw data supporting the conclusions of this article will be made available by the authors, without undue reservation.

## Ethics Statement

The studies involving human participants were reviewed and approved by the Washington University School of Medicine IRB University of California, San Diego IRB. The patients/participants provided their written informed consent to participate in this study.

## Author Contributions

AS designed the study, developed analysis tools, performed the analysis, and wrote the manuscript. TN performed the analysis, created all figures, and edited the manuscript. JS designed the study, developed analysis tools, and edited the manuscript. EL designed the study, secured funding, and edited the manuscript. JW designed the study and secured funding. MV organized the study working group. JM provided statistical expertise. MY performed statistical analyses. DM provided data archiving. JG provided essential data transfer expertise. JR analyzed the data. DS oversaw data acquisition at the San Diego site. DB designed the study and secured funding. LE oversaw and performed data acquisition at the San Diego site. All authors contributed to the article and approved the submitted version.

## Conflict of Interest

The authors declare that the research was conducted in the absence of any commercial or financial relationships that could be construed as a potential conflict of interest.

## Publisher’s Note

All claims expressed in this article are solely those of the authors and do not necessarily represent those of their affiliated organizations, or those of the publisher, the editors and the reviewers. Any product that may be evaluated in this article, or claim that may be made by its manufacturer, is not guaranteed or endorsed by the publisher.
