# Supplementary material for: Covariance and Correlation Analysis of Resting State Functional Magnetic Resonance Imaging Data Acquired in a Clinical Trial of Mindfulness-Based Stress Reduction and Exercise in Older Individuals
Source: Front Neurosci. 2022 Mar 18;16:825547. doi: 10.3389/fnins.2022.825547 (PMC8971902; doi:10.3389/fnins.2022.825547)
Supplement: Supplementary file 1 [file Data_Sheet_1.docx]

**Covariance and Correlation Analysis of Resting State Functional Magnetic Resonance Imaging Data Acquired in a Clinical Trial of Mindfulness-Based Stress Reduction and Exercise in Older Individuals**

This study was funded by NIH R01AG049689 by the National Institute of Aging together with the National Center for Complementary and Integrative Health, Office of Behavioral and Social Science Research, and the McKnight Brain Research Foundation. Additional support came from UL1TR002345 from the National Center for Advancing Translational Sciences. AZS was supported by NIH U19 AG032438, R01 AG072694-01A1, and 1P30NS098577. JSS and AZS were also supported by P50 HD103525 to the Intellectual and Developmental Disabilities Research Center at Washington University.

Supplemental Figures


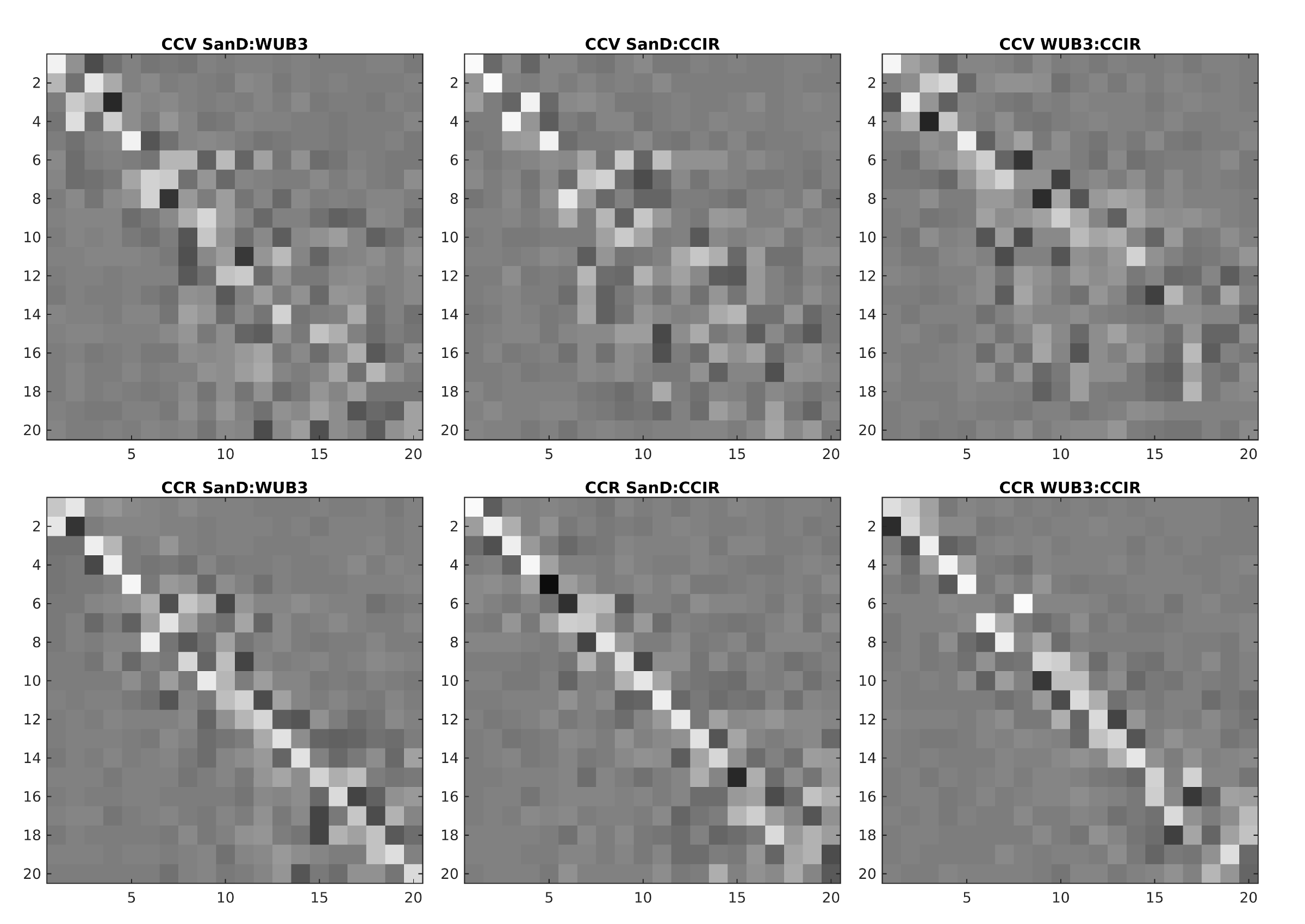
Figure S1. Basis similarity across scanners. POD of scanner-specific mean covariance and correlation matrices (main text Eqns. 2- 4) was computed to investigate dependence on scanner of basis vectors. Scanner-specific bases were not used in the reported analyses. The illustrated results show covariance (CCV) and correlation (CCR) matrix basis similarity displays corresponding to all possible scanner pairs. Thus, for example, "CCV SanD:WUB3" was computed as $\hat{W}_{SanD}^{T}\hat{W}_{WUB3}$ (see main text Table 3). Substantial scanner-dependent dis-similarity of covariance bases is evident, most likely attributable to imperfect gain-field inhomogeneity correction. Correlation bases appears to be relatively scanner-independent.


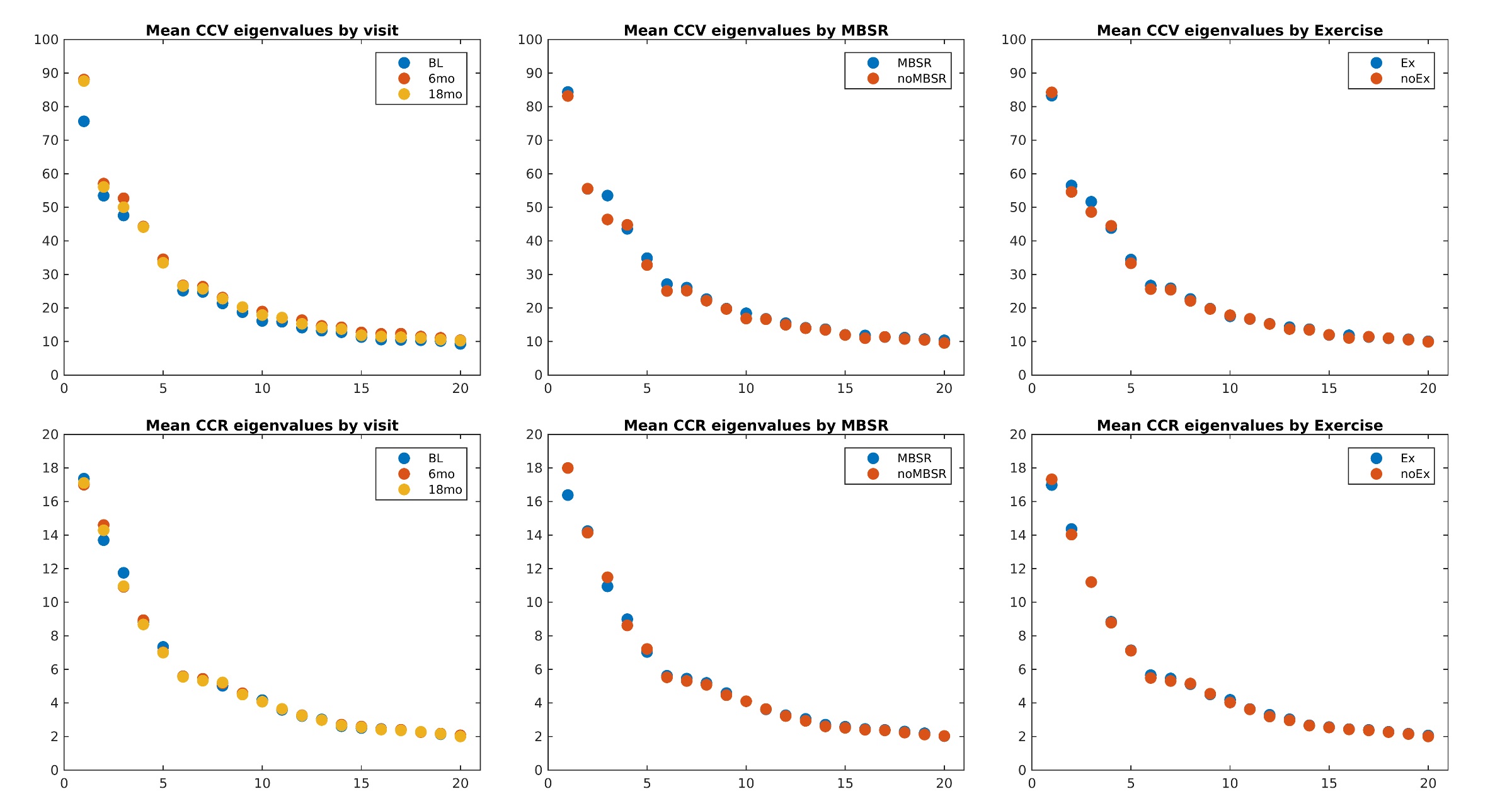
Figure S2. Scree plots broken down by subgroup. Individual covariance (CCV) matrices have been adjusted to ensure that total power (matrix trace) is identical across all three scanners. This operation does not apply to correlation (CCR) matrices.


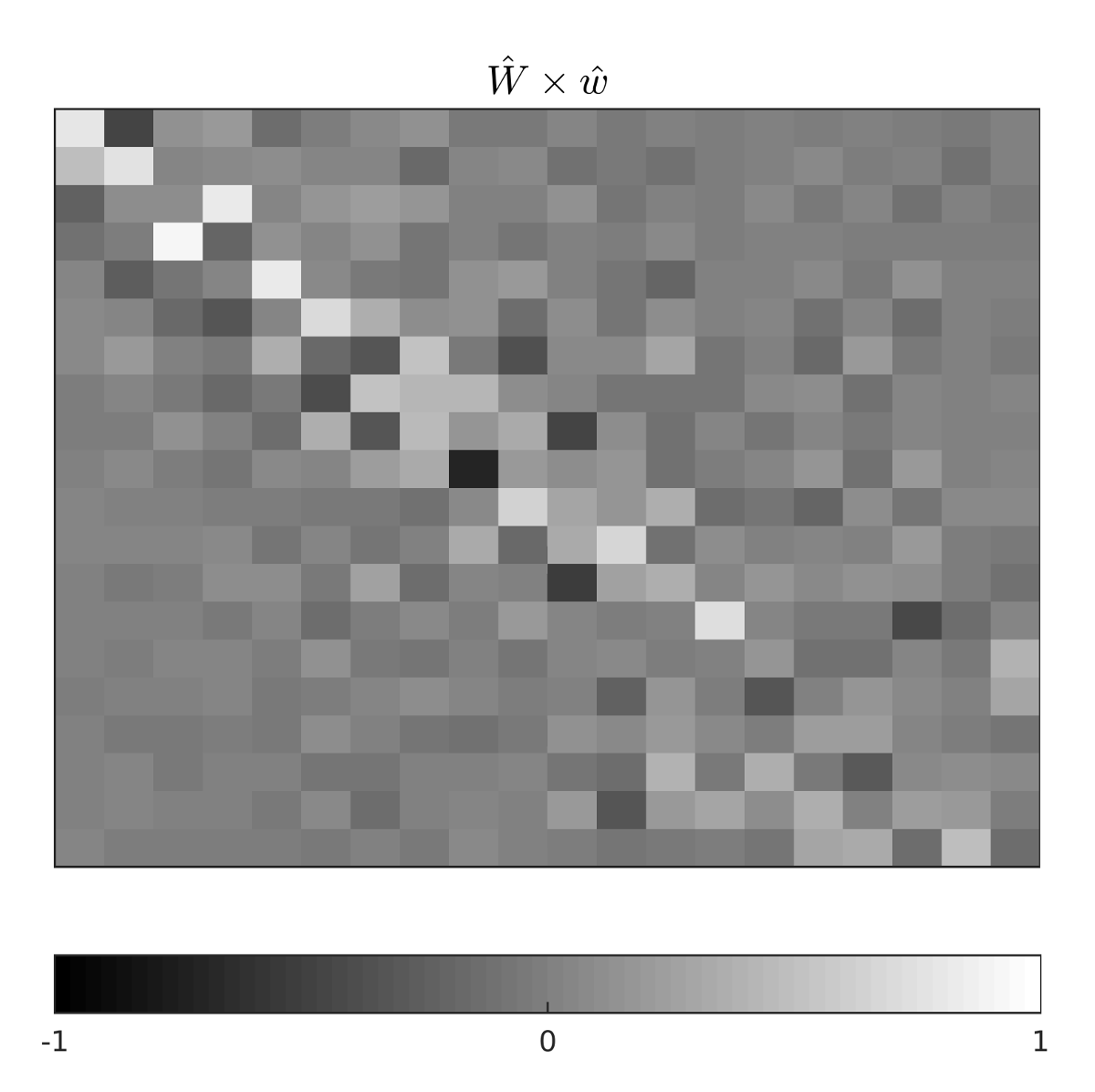
Figure S3. Similarity of covariance and correlation bases computed as $\hat{W}^{T}\hat{w}$. Perfect similarity would appear as the identity matrix (1 on diagonals, zero elsewhere). The actual result indicates modest reordering. Thus, for example, reordered bases 3 and 4 appear as bright (~1) values just off the diagonal. Additional comparison of $\hat{C}$ vs. $\hat{r}$ is shown in main text Figure 4.
